# Concept of the Munich/Augsburg Consortium Precision in Mental Health for the German Center of Mental Health

**DOI:** 10.3389/fpsyt.2022.815718

**Published:** 2022-03-04

**Authors:** Peter Falkai, Nikolaos Koutsouleris, Katja Bertsch, Mirko Bialas, Elisabeth Binder, Markus Bühner, Alena Buyx, Na Cai, Silvia Cappello, Thomas Ehring, Jochen Gensichen, Johannes Hamann, Alkomiet Hasan, Peter Henningsen, Stefan Leucht, Karl Heinz Möhrmann, Elisabeth Nagelstutz, Frank Padberg, Annette Peters, Lea Pfäffel, Daniela Reich-Erkelenz, Valentin Riedl, Daniel Rueckert, Andrea Schmitt, Gerd Schulte-Körne, Elfriede Scheuring, Thomas G. Schulze, Rudolf Starzengruber, Susanne Stier, Fabian J. Theis, Juliane Winkelmann, Wolfgang Wurst, Josef Priller

**Affiliations:** ^1^Department of Psychiatry and Psychotherapy, University Hospital, LMU Munich, Munich, Germany; ^2^Institute of Psychiatry, Psychology and Neuroscience, King’s College London, London, United Kingdom; ^3^Max Planck Institute of Psychiatry, Munich, Germany; ^4^Department of Psychology, LMU Munich, Munich, Germany; ^5^Münchner Psychiatrie-Erfahrene e.V., Munich, Germany; ^6^Institute of History and Ethics in Medicine, Technical University Munich, Munich, Germany; ^7^Helmholtz Pioneer Campus, Helmholtz Center Munich, Munich, Germany; ^8^Institute of General Medicine, LMU Munich, Munich, Germany; ^9^Department of Psychiatry and Psychotherapy, Technical University Munich, Munich, Germany; ^10^Department of Psychiatry, Psychotherapy and Psychosomatics, Medical Faculty, University of Augsburg, Augsburg, Germany; ^11^Department of Psychosomatic Medicine and Psychotherapy, Technical University Munich, Munich, Germany; ^12^Bundesverband der Angehörigen Psychisch Erkrankter Menschen, Bonn, Germany; ^13^BASTA – das Bündnis für Psychisch Erkrankte Menschen, Munich, Germany; ^14^Institute of Epidemiology, Helmholtz Center Munich, Munich, Germany; ^15^Institute of Psychiatric Phenomics and Genomics, University Hospital, LMU Munich, Munich, Germany; ^16^Neuroimaging Center, Technical University of Munich, Munich, Germany; ^17^Institute for AI and Informatics in Medicine, Technical University of Munich, Munich, Germany; ^18^Laboratory of Neuroscience (LIM 27), Institute of Psychiatry, University of São Paulo, São Paulo, Brazil; ^19^Department of Child and Adolescent Psychiatry, Psychosomatics, and Psychotherapy, University Hospital, LMU Munich, Munich, Germany; ^20^Oberbayerischen Selbsthilfe Psychiatrie-Erfahrener e.V., Altötting, Germany; ^21^Institute of Computational Biology, Helmholtz Center Munich, Munich, Germany; ^22^Institute of Human Genetics, Technical University Munich, Munich, Germany; ^23^Institute of Developmental Genetics, Helmholtz Center Munich, Munich, Germany; ^24^Department of Psychiatry and Psychotherapy, Charité – Universitätsmedizin Berlin, Berlin, Germany; ^25^Centre for Clinical Brain Sciences, UK Dementia Research Institute, The University of Edinburgh, Edinburgh, United Kingdom

**Keywords:** precision medicine, mortality, schizophrenia, depression, bipolar disorder, comorbidities

## Abstract

The Federal Ministry of Education and Research (BMBF) issued a call for a new nationwide research network on mental disorders, the German Center of Mental Health (DZPG). The Munich/Augsburg consortium was selected to participate as one of six partner sites with its concept “Precision in Mental Health (PriMe): Understanding, predicting, and preventing chronicity.” PriMe bundles interdisciplinary research from the Ludwig-Maximilians-University (LMU), Technical University of Munich (TUM), University of Augsburg (UniA), Helmholtz Center Munich (HMGU), and Max Planck Institute of Psychiatry (MPIP) and has a focus on schizophrenia (SZ), bipolar disorder (BPD), and major depressive disorder (MDD). PriMe takes a longitudinal perspective on these three disorders from the at-risk stage to the first-episode, relapsing, and chronic stages. These disorders pose a major health burden because in up to 50% of patients they cause untreatable residual symptoms, which lead to early social and vocational disability, comorbidities, and excess mortality. PriMe aims at reducing mortality on different levels, e.g., reducing death by psychiatric and somatic comorbidities, and will approach this goal by addressing interdisciplinary and cross-sector approaches across the lifespan. PriMe aims to add a precision medicine framework to the DZPG that will propel deeper understanding, more accurate prediction, and personalized prevention to prevent disease chronicity and mortality across mental illnesses. This framework is structured along the translational chain and will be used by PriMe to innovate the preventive and therapeutic management of SZ, BPD, and MDD from rural to urban areas and from patients in early disease stages to patients with long-term disease courses. Research will build on platforms that include one on model systems, one on the identification and validation of predictive markers, one on the development of novel multimodal treatments, one on the regulation and strengthening of the uptake and dissemination of personalized treatments, and finally one on testing of the clinical effectiveness, utility, and scalability of such personalized treatments. In accordance with the translational chain, PriMe’s expertise includes the ability to integrate understanding of bio-behavioral processes based on innovative models, to translate this knowledge into clinical practice and to promote user participation in mental health research and care.

## Introduction

Every year, approximately one third of adults in Germany and Europe meet the diagnostic criteria for a mental illness. Furthermore, 50% of adult mental health problems start before or during adolescence (1). Schizophrenia (SZ), bipolar disorder (BPD), and major depressive disorder (MDD) represent 12% of these illnesses [or 16% if clinical high-risk (CHR) states are included] and rank among the 10 most disabling diseases worldwide (2). Furthermore, in 2017 in Germany alone the costs of these diseases were €3.1 billion for SZ, €5.8 billion for BPD, and €8.7 billion for MDD (3). This burden results from chronic and/or relapsing syndromes that affect ∼50% of patients, i.e., 6% of the adult population, and include a wide spectrum of impairments, such as cognitive and negative symptoms (e.g., avolition, social withdrawal); affective symptoms, with depressive, dysphoric or elevated mood; persistent psychotic experiences (e.g., suspiciousness, persecutory ideas, and auditory hallucinations); and lasting psychosocial and vocational deficits (4, 5). Overall, there is a need for a deeper understanding, more accurate prediction, and personalized prevention of disease chronicity across these disorders and mental illnesses more broadly.

The six German Centers for Health Research (DZGs) carry out research on common diseases of particular importance for the health of the German population. The DZG were introduced to translate research findings more effectively into medical care and back (“From bench to bedside and back”). A new national research network on mental disorders, the German Center of Mental Health (DZPG), has now been established and is currently in a 6-month networking period to develop a coherent and complementary research program. In a review process, the Federal Ministry of Education and Research (BMBF) selected six partner sites of the DZPG, which include the universities of Berlin and Bochum, the Central Institute of Mental Health Mannheim/University of Heidelberg/University of Ulm, the universities of Jena/Halle/Magdeburg, and the universities of München/Augsburg, and Tübingen. The Munich/Augsburg site encompasses the Ludwig-Maximilian-University (LMU), Technical University of Munich (TUM), University of Augsburg (UniA), Helmholtz Center Munich (HMGU), and Max Planck Institute of Psychiatry (MPIP), which together form the research network “Precision in Mental Health (PriMe): Understanding, predicting, and preventing chronicity.”

**FIGURE 1:**
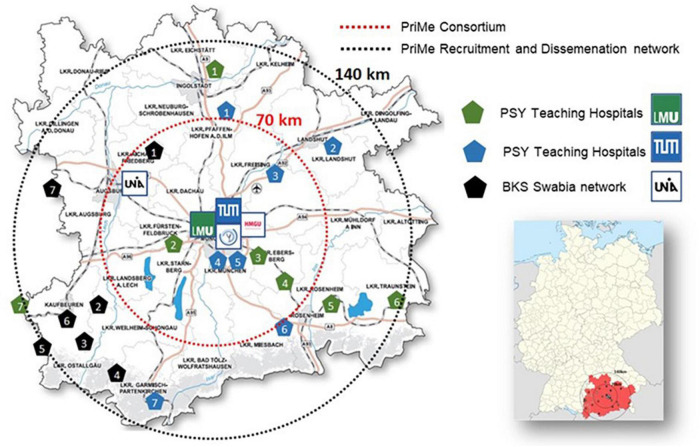
Map of the PRiME’s catchment area. PSY Teaching Hospitals LMU: 1. Klinikum Ingolstadt, 2. Hospital Fürstenfeldbruck, 3. Hospital München Ost, 4. Hospital Wasserburg, 5. Hospital Rosenheim, 6. Day Clinic Freilassing, 7. Hospital Memmingen. PSY Teaching Hospitals TUM: 1. Hospital Pfaffenhofen, 2. Hospital Landshut-Achdorf, 3. Hospital Freising, 4. Hospital Schwabing, 5. Hospital Bogenhausen, 6. Hospital Agatharied, 7. Hospital Garmisch-Partenkirchen. BKS Swabia network UniA: 1. Outpatient unit and day clinic Aichach, 2. Hospital Kaufbeuren, 3. Hospital Memmingen (allocated to two institutions), 4. Hospital Kempten, Day Clinic Lindau, 6. Hospital Obergünzburg, 7. Hospital Günzburg.

The Munich Metropolitan Area hosts an internationally acclaimed hub for mental health research with two excellence universities, the LMU and TUM; the UniA, with its newly established medical faculty; the HMGU; and the MPIP. This excellence builds on a long track record in bio-behavioral neuroscience, which originated in E Kraepelin’s and A Alzheimer’s seminal work on affective, psychotic, and neurodegenerative disorders and continues to thrive until today. Located in the Munich Metropolitan Area (26,000 km^2^), the PriMe consortium covers a catchment population of more than 6.2 million people, with 90,000 annual admissions (5,000 for SZ, 2,000 for BPD, and 22,000 MDD). The consortium extends to collaborating institutions, including outpatient and primary care networks (Figure 1). With its urban and rural regions, this catchment area is ideally suited for translational research from preclinical innovation across the clinical trial stages to final implementation in primary and resident specialist care.

In the following, PriMe’s scientific goals will be outlined by exemplifying some of the key contributions toward establishing a precision medicine framework for the Munich Metropolitan Area and the DZPG. To substantially improve patient outcomes, this framework will integrate better mechanistic understanding of pathogenesis and chronicity, individualized prediction, treatment and early intervention, as well as clinical implementation of disruptive therapeutic innovations across this value chain. In the subsequent chapters, we outline the specific need for early intervention and prevention in regards to SZ, BPD, as well as MDD and describe the novel scientific concept of the PriMe consortium including its preliminary work. Based on PriMe’s precision medicine framework, we present its five-part thematic focus. Furthermore, we illustrate PriMe’s infrastructure and its embedding in the German psychiatric research landscape. Moreover, we underline PriMe’s efforts in facilitating patient participation as well mentoring services for early career scientists. In addition, we discuss five platforms which will be implemented as part of the DZPG. These platforms are structured around the themes “Multimodal Data and Model Humanized Systems,” “Predictive Data Science,” “Personalized and Innovative Therapies,” Ethical, Societal, and Implementation Challenges” as well as “Clinical Trials and Evidence-Based Medicine.” We conclude by providing an outlook on PriMe’s activities contributing to an overarching program for the DZPG.

## Focus of PriMe on Early Intervention and Prevention of Chronicity and Mortality

**FIGURE 2:**
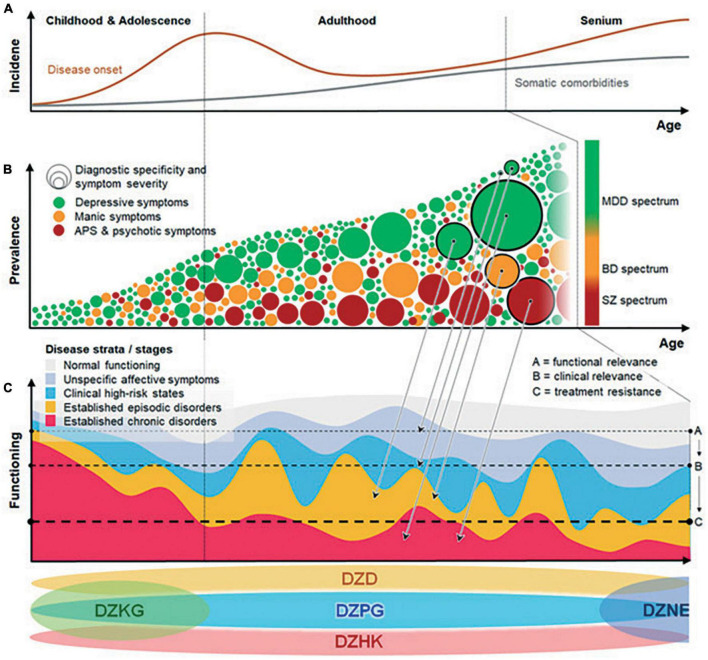
Concept figure illustrating the dynamics of mental disorders from a life-span perspective: **(A)** Incidence increases between adolescence and adulthood and toward old age. **(B)** Disease phenotypes evolve from non-specific to specific/severe manifestations. Arrows from panels **(B)** to **(C)** indicate how specificity/severity of phenotypes relates to disease strata. Heterogeneity increases as progressive and non-progressive trajectories, ranging from highly disabling phenotypes to minor impairments, mix at the population level. **(C)** Increasing functional impairment with disease progression from unspecific, at-risk to established episodic or chronic disease strata. At the bottom of the figure the positioning of different German Centres of Health covering mental healthcare-relevant topics across the lifespan are depicted. BPD, bipolar disorder; CHR, clinical high risk; DZD, German Center for Diabetes Research; DZHK, German Center for Cardiovascular Research; DZKJ, German Center for Child and Adolescent Health; DZPG, German Center for Mental Health; MDD, major depressive disorder; and SZ, schizophrenia.

Importantly, the risk for chronicity-related functional deficits is not confined to the established stages of SZ, BPD, and MDD but encompasses earlier, subthreshold mental conditions, commonly referred to as CHR states (Figure 2). In SZ, the attenuated psychotic or basic symptoms that characterize these CHR states mark a more specific and imminent risk for psychosis, and early precursors can be traced back to adolescence and even earlier. Generally, such precursors represent critical windows for early detection, prevention, and intervention strategies. For the last 15 years, PriMe members have established and maintained research and clinical infrastructures for the early identification of vulnerable individuals in at-risk and first-episode stages of SZ, BPD, and MDD, and PriMe aims to use these infrastructures to develop biomarker-informed, disease-interceptive treatments.

Mortality is markedly increased in SZ, BPD, and MDD, resulting in a mean of more than 10 years lost because of earlier death (6). Higher mortality is likely caused by complex interactions of risk factors, including psychiatric and somatic comorbidities, lifestyle-related factors, suicide and secondary illness/treatment effects (7). Furthermore, interactions between psychiatric illnesses, such as posttraumatic stress disorder (PTSD) and anxiety disorders, functional somatic syndromes, substance abuse, sleep disorders, neurodevelopmental conditions (e.g., autism), and personality disorders, also contribute directly or indirectly to this excess mortality. Detecting and treating such comorbidities in early SZ, BPD, and MDD may avert disease progression and promote clinical and functional recovery.

**FIGURE 3:**
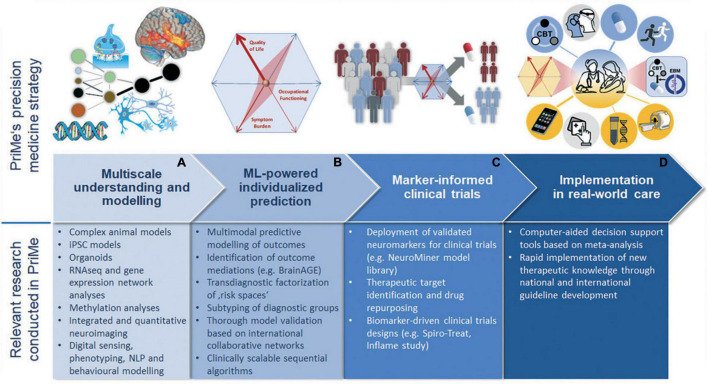
Concept figure representing the translational chain from understanding over development of predictive tools to their translation into clinical practice through stratified clinical trials **(A–D)**. The upper part depicts the envisaged bench-to-bedside sequence, while the lower part lists relevant research activities at each translational step that are being pursued by PriMe. iPSC, induced pluripotent stem cells; ML, machine learning; MRI, magnetic resonance imaging; NLP, neurolinguistic programming; NTBS, non-invasive transcranial brain stimulation; and PriMe, Precision in Mental Health.

Somatic comorbidities are hallmarks of SZ, BPD, and MDD that strongly contribute to the excess mortality of these system-level disorders. These comorbidities include metabolic illnesses, such as type II diabetes (T2D) and cardiovascular disorders (CVDs), and are main drivers of reduced life expectancy (8). They share pathophysiological (including immunological) pathways with mental disorders: For example, patients with T2D have an increased risk for MDD, and depressive syndromes worsen outcome in patients with myocardial infarction or stroke. PriMe aims at understanding, modeling, and treating the common roots of mental and somatic illnesses with a particular focus on immunological mechanisms (Figure 3). Often the aspects of somatic comorbidities in SZ, BPD, and MDD, i.e., their poor coverage, especially in primary care settings, are neglected. Particularly patients with depression and comorbid somatic disorders are cared for by primary care physicians (P). Similarly, pediatricians/family physicians are the first contact point for youths with psychosomatic syndromes, that may point to an increased risk for mental illness. Therefore, PriMe has teamed up with local networks of PCPs, pediatricians, and youth mental health services. PriMe also seeks close collaboration with the German Center for Child and Adolescent Health (DZKJ). At the other end of the life span, mental illness and somatic comorbidities represent risk factors for neurodegenerative diseases, which is why PriMe members collaborate closely with the local German Center for Neurodegenerative Diseases (DZNE) site. These networks will support the final step of the translational cascade, i.e., the implementation of easy-to-use clinical decision support tools in real-world care settings (Figure 3).

The seminal paper of the Lancet Commission (9) identified unhealthy diet and sedentary lifestyle as major risk factors for the physical illness burden of patients with SZ, BPD, and MDD. The Commission gives recommendations on how to influence this modifiable risk factor, e.g., including regular exercise in treatment plans. For the last 15 years, PriMe researchers have been working along the translational cascade (Figure 3) to better understand the neural effects of physical exercise (10) and to derive novel therapeutic interventions to improve patients’ capacity for neural plasticity, reintegration of body and mind functions, and functional recovery (11, 12), (WO/2006/120030).

Suicide risk and long-term psychopharmacological treatment also impact mortality rates. Population-based studies demonstrated that mortality can be reduced by more than 50% by effective antipsychotic treatment, especially when administered as a long-acting injection. PriMe researchers have acquired substantial expertise (Figure 3) in meta-analyses (13) of side-effect profiles of psychopharmacological treatments from the literature, making this knowledge available through computer-aided decision support tools (see the section “Prediction: Translating Understanding Into Precision Medicine Tools”). Furthermore, they have been involved in large-scale studies comparing the effectiveness and side-effect profiles of oral antipsychotics vs. long-acting injectables (13).

Lastly, the SARS-CoV-2 pandemic is challenging societies and health care systems, as well as the mental well-being of large parts of the population. In this context, a challenge for psychiatry is defining its role within the medical system, e.g., how psychiatric services can treat the mentally ill while supporting somatic medicine. To adapt patient services, the five PriMe institutions quickly established the following research structures: (1) epidemiological research (“MentalHealthCOVID-19” living systematic review on the consequences of the COVID-19 pandemic on mental health); (2) clinical research (online intervention programs for people with and without mental illness); (3) care structure research (Germany-wide survey on psychiatric care structures by the German Association for Psychiatry, Psychotherapy and Psychosomatics [DGPPN]); (4) nationwide surveys at UniA focusing on health care workers; and (5) biological research in close cooperation with the virology departments, e.g., LMU’s All Corona Care Study (ACC) on mental stress and resilience of health care workers (*N* = 8,000) and the involvement of LMU in the “EviPanUnimed” study within the BMBF-supported National research net-work of University Medicine on COVID-19 (NUM). TUM’s “MentalHealthCOVID-19” project is a living systematic review of epidemiological studies on the mental health consequences of the COVID-19 pandemic and related containment measures. The SARS-CoV-2–related research activities of PriMe members aim at collecting and analyzing multimodal data on the indirect effects of the pandemic in patients with SZ, BPD, and MDD and on the direct inflammatory effects that potentially interact with neurobiological risk factors for SZ, BPD, and MDD on a mid- to long-term basis.

## Novel Scientific Concept and Preliminary Work of the PriMe Consortium

PriMe aims to add a precision medicine framework to the DZPG that will propel deeper understanding, more accurate prediction, and personalized prevention of disease chronicity across mental illnesses. This framework will be structured into five methodological platforms that span the entire translational chain (Figure 3) and will be used by PriMe to innovate the management of SZ, BPD, and MDD from rural to urban areas and from patients in early disease stages to patients with long-term disease courses. PriMe’s goal is to establish precision medicine in mental health as the key clinical paradigm, allowing the development and implementation of personalized, preventive interventions for patients with SZ, BPD, and MDD. In accordance with the translational chain (Figure 3), the expertise stems from the ability to integrate understanding of bio-behavioral processes based on innovative models and translate this knowledge into clinical practice *via* predictive tools, novel treatments, and implementation strategies that follow the principles of open science. This expertise will enable groundbreaking innovations and clinical translation based on deeply phenotyped, representative cohorts of patients with SZ, BPD, and MDD, in addition to healthy individuals.

### Understanding and Identifying Genetic and Environmental Risk Factors for Unfavorable Disease Courses

**FIGURE 4:**
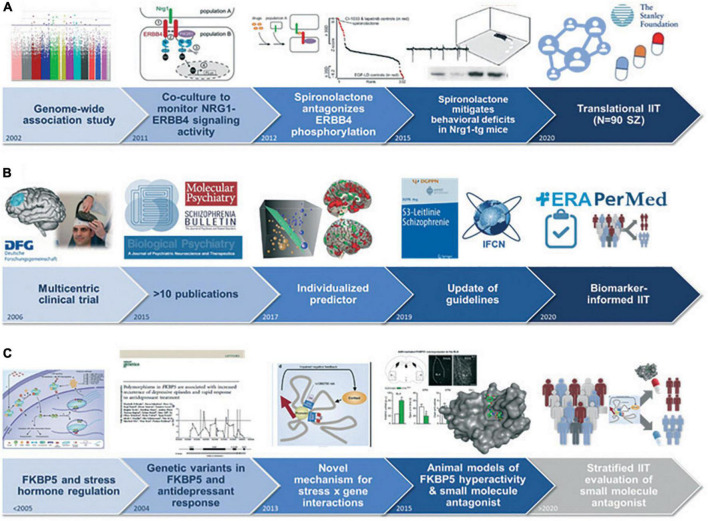
Use cases exemplifying the potential of biomarkers and risk prediction models for clinical translation: **(A)** Development and clinical evaluation of spironolactone as treatment for cognitive deficits in patients with schizophrenia (42). **(B)** Development and validation of an individualized structural magnetic resonance imaging-based rTMS response predictor for the targeted treatment of negative symptoms in schizophrenia (43). **(C)** Identification of a molecular mechanism of difficult-to-treat depression leading to the development of a small-molecule antagonist of FKBP5-induced disinhibition in rodents, with planned clinical translation (44, 45). IIT, investigator-initiated trial.

Both genetic and environmental risk factors moderate the development of SZ, BPD, and MDD across diagnostic boundaries. PriMe partners have delivered important insights into genetic disease mechanisms *via* genome-wide association studies (GWASs) and large-scale sequencing approaches, taking leading roles in genomic consortia (ConLiGen, Restless Legs syndrome and Dystonia, the Psychiatric Genomics Consortium, and CLOZIN) and methods development. The first GWAS on lithium response, the identification of *MEIS1* as a major risk gene in restless leg syndrome, contributions to the pharmacogenetics of antidepressant response, and the genetic architecture of MDD (14) are only a few high-impact examples published by PriMe researchers. To improve mechanistic insights, these approaches have been extended to omics layers, from transcriptomics and epigenomics to metabolomics, with a focus on single-cell resolution. At the environmental level, early adverse life experiences constitute major risk factors for unfavorable courses of SZ, BPD, and MDD. PriMe members have been striving to understand how these experiences act upon risk genes *via* dysregulation of molecular, cellular, brain-based, systemic, and social network functions (gene × environment interactions) that lead to the biological embedding of psychological trauma and ultimately poor disease outcomes (Figure 4). PriMe partners described the first molecular mechanism of gene × childhood trauma interactions linked to FK506 binding protein 5 (FKBP5) (15). FKBP5 acts as an endogenous regulator of the stress-neuroendocrine system and plays an important role in neurons and immune cells and thus constitutes a risk factor in stress-related disorders. FKBP5 also provides a starting point for uncovering signatures of childhood adversity on molecular (mainly epigenetic), neuroanatomical, endocrine, psychophysiological, functional, and psychosocial levels. These findings stimulated pharmacological innovations and new research on trauma-focused interventions.

### Trauma-Related Gene × Environment Interactions and Neuroimmune Mechanisms Moderate Neurodevelopment From the Womb to Early Adulthood

A better longitudinal understanding of exposure and bio-behavioral risk patterns in any given patient will personalize prevention and treatment strategies more precisely than current cross-sectional high-risk detection approaches. To this end, PriMe researchers have reported how (1) pre- and perinatal risk factors influence brain development so that their brain structural, functional, and neurocognitive impacts can be traced into adulthood (e.g., Developing Human Connectome Project, Bavarian Longitudinal Study of Prematurity); (2) epigenetic changes after exposure to prenatal adversity may contribute to the risk for psychiatric disorders; and (3) polygenic risk markers, in combination with measures of childhood trauma, predict depression and psychosis onset in adolescence and early adulthood (16).

Environmental risk factors may also act on molecular or brain pathology *via* known psychosocial, psychological, and neurocognitive mechanisms. PriMe has accumulated evidence that personality traits (low extraversion and high neuroticism and anxiety), impaired neurocognitive functions (low cognitive control and processing speed), dysfunctional coping styles (repetitive negative thinking, dysfunctional emotion regulation), interpersonal dysregulation (insecure or disorganized attachment, impaired coping with social exclusion), and psychosocial risk factors (low social support and socioeconomic status, parental psychopathology, and migration status) entail unfavorable disease outcomes (17).

An important mode of action of these factors on disease pathology and the course of SZ, BPD, and MDD is the activation of neuroimmune mechanisms within critical maturational windows. Prenatal immune activation, e.g., by maternal infection during pregnancy, acts synergistically with exposure to postnatal trauma to induce long-lasting neurochemical and behavioral disturbances. Microglia are innate immune cells that colonize the developing brain and sense pathological changes in the central nervous system (CNS) (18). They are crucial for neural plasticity and the maintenance of brain homeostasis but can be primed for innate immune memory. Recently, PriMe members provided the first single-cell analysis of human microglia in health and MDD (German Research Foundation CRC/TRR167), revealing disease-associated states that may be exploited for therapeutic purposes (19). PriMe investigators have also contributed important new leads in the description of autoimmune mechanisms that underlie forms of psychosis that are responsive to immunomodulatory therapies.

### Prediction: Translating Understanding Into Precision Medicine Tools

The ability to predict disease courses, treatment responses, and relapse patterns of any given patient is the pacemaker of personalized medicine. Individualized prediction depends on quantitative models that integrate mechanistic knowledge with clinical, psychological, psychosocial, environmental, and biological disease markers. These multi-modal analytical approaches rest on representative longitudinal patient cohorts that cover the cross-sectional and outcome-related heterogeneity of the target population and facilitate the prediction of short-term treatment responses to long-term disease outcomes. PriMe members have successfully established, coordinated, and analyzed such cohorts over the last 20 years, including the EU-funded PRONIA study (*N* = 1,976), which is currently powering the development of precision medicine tools for patients with early stage affective and psychotic disorders (20), and the PsyCourse study (*N* = 1,303), which aims to decompose the heterogeneity of psychotic and affective syndromes into distinct trajectories and patient subtypes (16). Furthermore, antidepressant treatment response was longitudinally characterized within the MARS study in 1,400 patients to develop depression-specific outcome predictors and disease course models (21). The BeCOME study (*N* > 350, recruitment ongoing) expands this concept to a transdiagnostic approach, which aims at identifying disease domains through multilevel deep phenotyping, thus contributing to a new and more precise taxonomy for affective syndromes.

High-quality longitudinal samples of patients covering the three target conditions are essential but not sufficient for developing tools that enable more precise mental health care strategies, and cutting-edge methods in predictive data science are equally important. PriMe members are pioneers in psychiatric machine learning (ML) and have showed that clustering patients with established psychosis reveals disease subtypes with distinct disease courses and genetic underpinnings (17). They developed novel methods to efficiently combine diverse phenotypic and biological model predictions in individual patients and observed that such algorithms enable the clinically scalable prediction of poor outcomes in at-risk states for psychosis or recent-onset depression (20). Multi-site clinical trial data showed that it is equally possible to establish models for predicting response to non-invasive transcranial brain stimulation (NTBS) or antipsychotic treatments. These achievements were facilitated by the ML platform NeuroMiner,^1^ which has been developed by PriMe partners since 2009 and provides clinician neuroscientists with openly accessible algorithms for robust model construction, validation, and knowledge sharing. PriMe has also spearheaded deep learning in large-scale population studies, e.g., ML-derived decision support based on neuroimaging (22). Pushing these modeling approaches closer to their clinical implementation, PriMe members have established an ML Model Library^2^ that provides the research community with access to published models for external validation and experimental clinical use.

Furthermore, the in-depth analysis of multimodal, large-scale samples planned by PriMe (see the section “Innovative Therapies to Preempt Disease Progression and Chronicity”) requires novel computational approaches beyond classical ML. These approaches involve deep learning that can integrate multiple layers of biological and phenotypic measurements, including high-frequency sensor-based assessments, to identify clinically useful predictive signatures. These technologies enable analysis of high-throughput data as generated by ecological momentary assessments (EMA), digital sensing, and natural language processing and thus hold great promise for novel diagnostic and therapeutic applications. PriMe members developed mobile sensing technology to continuously collect and analyze these novel information domains, including measures of mobility, smartphone interaction behavior, speech and verbal synchrony, facial expressions, eye tracking, and keyboard usage (23).

Furthermore, PriMe members aim to digitize and standardize the routine collection, integration, safe processing, and redistribution of clinical information to establish personalized, measurement-based care in psychiatry. All these efforts are supported by cutting-edge tools for federated and privacy-preserving ML and guided by ethical standards for medical innovation and health technologies. The Munich School for Data Science and the German Human Genome-Phenome Archive will contribute significant expertise in embedding ethical artificial intelligence (AI) approaches in the new technologies.

A critical link between animal models and individualized prediction approaches is established by means of psychiatric neuroimaging. PriMe members have initiated standardized imaging protocols across all their scanner facilities and participate as neuroimaging partners in a German-wide initiative to standardize neuroscience data for open science and data sharing (NFDI-Neuro). This includes expertise in the development of novel multimodal imaging techniques and their evaluation in neuropsychiatric patients, including the characterization of functional and structural brain network profiles in patients with SZ, BPD, and MDD. In a recent study, the effect of brain stimulation on these brain networks was optimized for application in individual patients, and biophysical modeling of excitatory and inhibitory neurotransmitter signaling revealed region-specific stimulation effects. Moreover, PriMe partners have developed methods for cutting-edge analysis of broadly available and cost-effective electroencephalogram (EEG) recordings for the standardized assessment of brain oscillations and synchrony in psychiatric disorders. PriMe members at TUM have also pioneered quantitative metabolic and functional imaging of the human brain that provides novel insights into the neuroenergetics and directional signaling of large-scale brain networks (24). This research has been further extended to incorporate the dynamics of neurotransmitter signaling in relation to classical brain network imaging. This technology has been used to describe trajectories of aberrant dopamine transmission and their impact on brain network activity in the course of psychotic disorders and characterized the dynamics of gamma aminobutyric acid/glutamate signaling in relation to brain connectivity in the healthy brain (25).

### Innovative Therapies to Preempt Disease Progression and Chronicity

PriMe identification strategies aim at personalizing available interventions, developing novel treatments, and clinically combining both to prevent disease progression, recurrence, chronicity, and mortality. Thus, disease-relevant model systems and readouts with translational relevance need to be established, a process that is especially challenging for psychiatric disorders (Figure 4). Induced pluripotent stem cells (iPSCs) from individuals with SZ, BPD, and MDD have opened avenues to study human neurodevelopment, polygenic risk, and neuro-immune interactions. PriMe members have established and shared more than 100 iPSC lines from patients treated at LMU and MPIP. Research conducted with iPSC-derived neurons and glia, including microglia, and cerebral organoids as neurodevelopmental models (26) showed their translational potential for investigating molecular and cellular mechanisms of treatment response. PriMe also hosts the German Mouse Clinic (GMC), which includes an open-access phenotyping platform for the comprehensive characterization of mouse models of human diseases and for preclinical drug testing. PriMe has thus established pathways for forward and reverse translation to facilitate the development of innovative and targeted treatments.

In neuropsychopharmacology, PriMe has envisaged (1) hit-to-lead approaches, in collaboration with partners such as the Lead Discovery Center in Dortmund, and several hits emerging from human genetic and basic research findings are now being studied, including new targets for MDD (FKBP5, SLC6A15, Beclin1, and Erythropoietin variants); and (2) drug repurposing, where preclinical evidence from cellular and animal models puts the focus on the neuregulin-1 (NRG1)-ErbB4 pathway, which, as a regulator of the excitatory/inhibitory disbalance in SZ, likely contributes to residual cognitive symptoms: In a large drug screen conducted by LMU, spironolactone emerged as a pathway modulator (27), leading to a pioneering and recently completed multicenter Investigator-Initiated Trial (Figure 4).

PriMe additionally focuses on brain circuits and networks as targets for NTBS. LMU researchers established and coordinate the German Center for Brain Stimulation (GCBS) Consortium within the BMBF-funded German Research Network for Mental Disorders, an internationally visible hub for translational research on NTBS (with >30 publications during the funding period) aimed at developing innovative NTBS-based treatment strategies for psychiatric disorders. Moreover, together with the GCBS, PriMe partners are conducting various translational studies (28) and randomized controlled trials (RCTs) for the treatment of SZ and MDD that are funded by the DFG, BMBF, and EU (ERA-NET NEURON and ERA PerMed). For the region of South Bavaria, neurostimulation centers at LMU and UniA collaborate with NTBS groups at the TUM and provide novel NTBS methods and protocols to patients with SZ and MDD with a fast translational track from discovery to clinical application.

PriMe further explores other novel treatments, such as exercise therapy (ET), and is performing the largest ongoing RCT on ET in SZ (BMBF-funded ESPRIT Consortium) (29). In the field of psychotherapy, PriMe partners, funded by the DFG, BMBF, EU, and Innovationsfond, develop novel mechanism-based therapeutic strategies, including (1) trauma-focused interventions for disorders related to early adversity, bodily distress, and cognitive bias; (2) transdiagnostic interventions for depression and anxiety disorders; (3) interpersonal therapies for persistent depressive disorder; (4) interventions tailored to vulnerable and underserved groups (e.g., refugees); (5) emotion regulation training for young patients with MDD; and (6) family based programs aimed at preventing adolescent depression in at-risk cohorts. PriMe has also conducted proof-of-concept psychotherapy studies targeting empirically established biobehavioral mechanisms and including pre-post neuroimaging for identifying neurobiological markers of change (KFO 256). In addition, PriMe members have tested novel treatments for the interactions of bodily symptoms (including pain) and depression in oncology and neurology (German Cancer Aid, BMBF IFB Dizziness). These studies integrate concepts of acute and long-term treatment, aim at potential stratifiers for future interventions and leverage the development of psychobiological markers. These strategies are also adaptive to acute societal needs: As thousands of refugees a day arrived in Munich in 2015, PriMe coordinated medical and psychological emergency care and, together with other centers, tested a stepped care approach in psychotherapeutic interventions for these vulnerable persons (MEHIRA) (30).

### Implementation: From Synthesis of Current Knowledge to Guidelines and Knowledge Transfer

PriMe has internationally recognized expertise in the field of evidence-based mental health care, spearheaded by the schizophrenia guideline groups [Association of the Scientific Medical Societies in Germany (AWMF) evidence and consensus-based S3 guidelines, World Federation of Societies of Biological Psychiatry guidelines, and International College of Neuropsychopharmacology guidelines for SZ] and the Section on Evidence-Based Medicine (EBM) in Psychiatry and Psychotherapy. The section now leads the Cochrane Schizophrenia Group and excels in all types of systematic reviews (Figure 4). PriMe will set new standards in methodology and translate results into novel therapeutic decision support tools. Examples are “living” (MentalHealthCOVID-19) and crowdsourcing-based systematic reviews on the mental health consequences of the COVID-19 pandemic; the EVENT study, which translates meta-analytic data (31) into digital decision aids for patients and professionals; and the cooperative SISYPHOS project, which is developing the first living guideline in German mental health care for digital and individualized treatment decisions.

PriMe researchers greatly value the principles of reproducibility and open science. To this end, the biobanks of PriMe’s institutions pertain to the highest quality standards for ascertaining clinical phenotyping and biological data on genetics, proteomics, neuroimaging, and neurophysiology. PriMe researchers follow the TUM Open Access Policy or are members of LMU’s Open Science Center (OSC) and Open Science In Medicine (OSIM) initiative. These programs are led by LMU Psychology and supported by a DFG priority research program focusing on the reproducibility of scientific findings (32). Since 2019, they have been sharing raw data and scripts from published articles (33, 34) and providing data for large multi-site analyses (32). To encourage reproducible data science, PriMe members have also established a public-access library hosting all published NeuroMiner models and Neuromodulation and Multimodal NeuroImaging software (NAMNIs) (YZEpLGDMJM0),^3^ a standardized software that facilitates replicability of results. Predictive, personalized, and technology-based medicine can come with ethical and societal challenges, e.g., algorithmic bias, impact on patient autonomy, social stigmatization, and loss of quality of life (35), and PriMe will address these issues with an interdisciplinary and flexible ethics approach which aims to recognize and address ethical and social issues as they emerge during innovation and translation.

## Infrastructure of PriMe Including User Involvement and Young Scientists

PriMe’s translational mindset evolved through an array of national and international research consortia, centers of excellence, several graduate schools, a clear commitment to all aspects of evidence-based psychiatry, and a broad array of implementation approaches. PriMe members broadened the scope of these approaches from the classical “bench-to-bedside” paradigm to concepts of forward and reverse translation and “bench-to-individualized guideline” strategies. These structures not only permit a seamless translation of novel preclinical approaches to clinical care but also enable a reverse, “guideline-to-bench” translation. PriMe fully supports translational research in the DZPG with infrastructures that cover the entire translational chain. These infrastructures include outstanding platforms for the identification of risk factors (multi-omics technologies), the production and analysis of predictive human cellular and humanized animal models, drug discovery, highly specialized imaging tools, centralized biobanks, comprehensive patient cohorts, and an extensive network of clinical trial centers led by the Munich Study Center (MSZ). PriMe’s specific contributions to DZPG research hubs include the central biorepository and datasets of the German National Cohort study (NAKO Gesundheitsstudie), the open-access German Mouse Clinic (GMC), the outstanding neuroimmunology hub (e.g., SyNergy Excellence cluster), the Leibniz Supercomputing Center (LRZ), strong artificial intelligence (AI)ML analysis platforms, NTBS facilities, comprehensive clinical trial and patient-recruitment infrastructures, and excellence in EBM *via* a Cochrane Review Group. Built in 2001, the GMC was the first platform worldwide (36) for systematic phenotyping and has advanced to a well-established and unique center for state-of-the-art mouse phenotyping. The GMC is an open-access phenotyping platform characterizing mouse models for human diseases in multiple body systems and physiological pathways in a variety of therapeutic areas. Its collaboration with more than 170 groups has resulted in more than 200 highly cited publications on neurodevelopmental and disorder-related phenotypes, advances in modeling abnormal neurodegenerative and aging patterns [with the German Center for Neurodegenerative Diseases (DZNE)], and diabetes models (with the German Center for Diabetes Research [DZD]). Founded in 1962, the LRZ is the IT service provider for PriMe partners and serves as a hub for other research organizations in Bavaria. LRZ is located in the Munich Metropolitan Area and is one of the three national members of the Gauss Center for Supercomputing, the High-Performance Computing Center Stuttgart (HLRS), and the Jülich Supercomputing Center. Helmholtz AI has established a network of method specialists (AI consultants) for short- and mid-term collaborations. The Helmholtz AI computing resources are available for all collaborative projects on identifying biomarkers and patient groups at risk for chronicity. The TUM Institute for AI and Informatics in Medicine (AIIM@TUM) is leading DIFUTURE, one of the four consortia funded by the BMBF, during the development and networking phase of the Medical Informatics Initiative. Its infrastructure and AI expertise will support the advanced analysis of preclinical and clinical data in a secure, federated, and privacy-preserving manner.

NAKO (37) (since 2014, *N* = 20 500, ages: 19–69), KORA (since 1984, *N* = 18 000, ages: 25–74), and GINIplus and LISA (since 1995, *N* = 9,085; see the section “Prediction: Translating Understanding Into Precision Medicine Tools”) are longitudinal cohort studies collecting information on mental health (e.g., depression, anxiety, PTSD, cognitive impairment, sleep disorders, subjective health, well-being), lifestyle (e.g., smoking, physical activity, alcohol, and body mass index), comorbidities (myocardial infarction, stroke, T2D, cancer), sociodemographics (socioeconomic status, household size), and bio-samples, multi-omics data, and whole-body MRI. Specifically, the GINIplus and LISA studies provide unique opportunities to investigate how early life events affect long-term mental health development. Both studies recruited multi-site birth cohorts comprising healthy, term-born infants from the general population and have been regularly following these cohorts (38). In close collaboration with PriMe members, age-appropriate mental health assessments have been integrated during the 10-year follow-up, which will enable PriMe and the DZPG to identify risk factors for disease progression, recurrence, and chronicity. The cooperation of PriMe partners in KORA, will allow disease course and outcome data on the available cohorts to be added.

Furthermore, PriMe has access to deeply phenotyped, longitudinal cohorts, enabling the study of risk factors of poor outcomes of CHR (PRONIA) and first-episode (PRONIA, BeCOME) and multi-episode disease stages (PsyCourse, BeCOME) in SZ, BPD, and MDD. PRONIA is a European sample of 1,400 patients with CHR states, first-episode depression, and psychosis that provides clinical, neuropsychological, imaging, proteomic, inflammatory, and genetic data. It is involved in the NIMH-funded HARMONY consortium, which aims at thoroughly validating predictors of disease outcome in CHR states of psychoses. PsyCourse includes 1,303 patients with multi-episode SZ and BPD, 800 of whom were followed up at four different times (17). BeCOME is a prospective, ongoing, transdiagnostic cohort focusing on affective and anxiety disorders. The goal is to characterize underlying multilevel biological trajectories of affective syndromes. BioMD-Y collects genetic and clinical data and comprehensive information on childhood trauma and other adverse life experiences in a longitudinal cohort of patients with adolescent depression (*N* = 420). PriMe institutions and the 32 teaching hospitals and integrated community hospitals in the Munich Metropolitan Area have implemented a powerful recruitment network to conduct large-scale clinical trials based on the aforementioned interventional approaches. This *trans*-sectorial recruitment approach is enhanced by a recruitment collaboration with an extended network of resident specialists (*N* = 270) and primary care physicians (*N* = 400) with 1.04 million patient contacts/year. Thus, PriMe covers all relevant sectors of the local health care system and integrates both urban and rural care environments.

Implementation of research involving service users is urgently needed. Specifically for PriMe, existing participative research approaches have been combined to form the “Munich working group for participatory research within the DZPG,” which now involves people from most stakeholder groups (patient and caregiver representatives) in the greater Munich area and has already contributed to the current application. This working group will serve as an advisor for DZPG research projects with regard to participatory research. In addition, it will initiate user-led research projects. Hereby, the working group can build upon existing participatory collaborations, such as the EVENT project, which embeds meta-analytic knowledge on antipsychotic drugs effects in an app to stimulate shared decision-making between patients and clinicians, or a recent study on the inclusion of caregivers in clinical decision-making (39).

Regarding implementation research, PriMe members have a long-standing track record in this field. Among many others, three projects (SISYPHUS, the MUNICH model, and RETURN) demonstrate the wide spectrum of efforts in this area: (1) The SISYPHOS project will test two strategies for implementing the German national schizophrenia guideline across 16 psychiatric hospitals. (2) The Munich Model is a disease management program that has been successfully implemented by TUM since 2006; it incorporates all evidence-based treatments and strategies related to relapse prevention in SZ and MDD, such as personal compliance profiles, psychoeducation, depot treatment, and others, and its success has been demonstrated (40). (3) The RETURN study focusses on how people with mental illness can best be supported when returning to their workplaces after being treated in a psychiatric hospital (41).

PriMe offers infrastructures for the training of medical students, nurses, psychologists, and medical specialists toward primary care, psychiatry and psychotherapy, and psychosomatic medicine and of clinician scientists. Examples is new master’s course in psychotherapy at LMU Psychology in collaboration with LMU Psychiatry; the Medical Education Centers at LMU and TUM; the “Hausarzt 360” program, with its large network of teaching practices; and the new DFG-funded Graduate School “Predictors and outcomes in primary depression care” (POKAL). The PriMe initiative will offer internationally competitive education to early career researchers (ECRs) through structured graduate programs in neuroscience and psychiatric research. These programs, such as the International Max-Planck Research School Translational Psychiatry (IMPRS-TP) and the Else Kröner-Fresenius College “Translational Psychiatry,” build on existing infrastructures for career development with an international perspective. Courses, seminars, lectures, Thesis Advisory Committees, and our internationally staffed research groups communicate in English. International Ph.D. students and postdocs serve as role models for ECRs with an international background. Attracting and developing young, high-potential scientists are important aspects of academic competition. Systematic mentoring is key in this respect and addressed by university programs.

## Discussion

A disease stage-sensitive prevention approach is needed to reduce the disabling manifestations of SZ, BPD, and MDD. As previously shown, prevention is most effective during active biobehavioral maturation phases, i.e., typically at the junction between adolescence and early adulthood (Figure 2). Detecting vulnerable individuals in these stages is challenging given the low temporal stability and low diagnostic specificity of symptom patterns (Figure 2). Hence, powerful yet broadly accessible diagnostic, prognostic, and therapeutic tools are needed to precisely identify and preventively treat persons at risk for poor disease courses. At this stage, preventive treatments should not only aim at reducing symptom burden but also strengthen the person’s resilience against adverse outcomes and follow well-established concepts of primary prevention for children and adolescents. However, such preventive approaches will not completely avert clinical and functional deterioration in vulnerable populations. Therefore, new stage-sensitive therapies are needed for patients with established disorders to reduce symptoms, disability and comorbidities and activate recovery with risk-informed plasticity-enhancing interventions. IT-powered mental health networks distributed across the patient’s local network of mental healthcare providers are indispensable for tailoring treatments to the patient’s needs and covering the critical windows in a person’s life in an ongoing and participatory fashion. Finally, similar concepts apply to the increased risk for neurodegenerative disorders, which are frequently preceded by affective syndromes during the transition to old age (Figure 2). PriMe will engage with the DZKJ and the DZNE to address these critical time windows synergistically.

**FIGURE 5:**
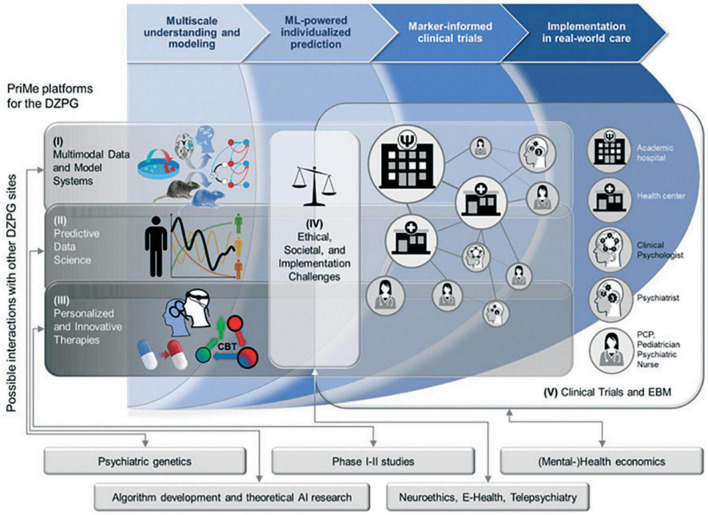
Mapping between PRiME’s translational chain and envisaged research platform in the new German Centre for Mental Health (DZPG). These platforms will be generic and thus accessible by all sites of the DZPG to facilitate the rapid translation of knowledge into therapies for all mental disorders. Boxes represent topics where PriMe could benefit from the expertise of other potential DZPG sites. AI, artificial intelligence; DZPG, German Center for Mental Health; EBM, evidence-based medicine; and ML, machine learning.

In Germany, major roadblocks currently exist on the path to mental health care approach because the national health care system is primarily focused on the management of manifest and chronic conditions. This approach largely dismisses a trajectorial concept of mental disorders and wastes opportunities for earlier, more preventive and less burdensome interventions. A downstream effect is that mental health care services lack a unified digital backbone that would integrate and organize the multi-faceted health-related data produced by the streams of diagnostic and therapeutic processes. Building clinical networks on such an infrastructure (Figure 5) not only allows patients to be more effectively treated by the connected stakeholders of a local healthcare system but also expedites the development and transfer of tools and treatments to care according to a bidirectional process. By making data available that represent disease variability across all stages of SZ, BPD, and MDD, clinical networks would facilitate research into the mechanistic understanding, predictive modeling, and development of personalized therapeutics (Figure 4). At the end of this translational chain, clinical networks would quickly harness the results of well-powered clinical trials that test the effectiveness of personalized therapeutic strategies with measurable markers. Based on these concepts, PriMe’s objectives in the DZPG areto develop, test and implement an early, preventive, stepped-care approach to the treatment of SZ-, BPD-, and MDD-spectrum disorders and their most prevalent comorbidities in the DZPG to continuously support all DZPG sites to do likewise for other mental disorders (e.g., addiction, anxiety disorders) by enabling multisite projects to develop strategies for any disease phenotype with the standardized toolkits of PriMe’s 5 interconnected platforms (Figure 5) and by validating components of these strategies retrospectively in PriMe’s existing large-scale cohorts and prospectively in PriMe’s deeply characterizable and representative catchment population (Figure 1). Further information on these five platforms is provided below:

### Platform I: Multimodal Data and Model Humanized Systems

This platform will focus on biobanking, neuroimmunology, stem cells, and multi-omics (Figure 5) and will be powered by the centrally managed, high-capacity biobanks of PriMe partners, providing harmonized recruitment and phenotyping standard operating procedures for all patient admissions to the mental health services of PriMe. This PriMe Biobank network will enable other sites to safely process, distribute and analyze biological specimens and patient-derived induced pluripotent stem cells (iPSCs) on demand across the DZPG. The Neuroimmunology hub and Munich Sequencing Alliance (see the section “Innovative Therapies to Preempt Disease Progression and Chronicity”) will use this harmonized biomaterial repository to produce multi-omics data for complex analytical endeavors and clinical applications across the DZPG. Harmonized phenotyping and (single-cell) multi-omics approaches that can be further expanded nationally and internationally will be critical for obtaining the sample sizes necessary for detecting relationships between risk factors, symptoms, and diseases.

To facilitate forward and backward translation of pathophysiological knowledge between iPSCs, animals, and humans, cutting-edge multimodal imaging is planned (e.g., simultaneous dopamine-PET/functional MRI/EEG in humans complemented by sub-second multi-site dopamine imaging in behaving mice). MRI, PET, and EEG protocols and data acquisition at the research-dedicated PriMe imaging facilities will be harmonized and supported by a computational backbone to store, preprocess and distribute imaging data. This imaging repository will be integrated into the biobank to make brain descriptors of mental disorders readily available for downstream analyses.

Behavioral indicators of disease progression risk and chronicity will be assessed with innovative digital approaches (smartphone-based EMA, mobile sensing, natural language processing). These digital markers of normal and pathological behavior will be safely stored and processed in PriMe’s extended biobank repository, thus enabling further modeling and predictive and therapeutic research in the DZPG. By using available blood and imaging-based markers and environmental and behavioral variables, PriMe will enable the DZPG to produce and harmonize cellular (iPSCs, organoids) and animal models reflecting the pathophysiology of unfavorable courses in mental disorders.

### Platform II: Predictive Data Science

PriMe proposes an ML-based data analytics platform as a core transdiagnostic facility of the DZPG. The platform will bundle clinical domain knowledge and applied engineering to derive predictive models from representative datasets, enabling the early identification of patients at risk of disease progression and the modeling of common comorbidities by using multi-view and transfer learning based on the KORA, NAKO, and GINIplus and LISA samples. The platform will be jointly run by (1) the new TUM Institute for AI and Informatics in Medicine, focusing on advanced neuroimaging and ML methods in collaboration with the new Neural Engineering for Mental Health network at TUM; (2) the HMGU Institute of Computational Biology, contributing multivariate genomic data analysis and deep-learning–based integration with clinical covariates; and (3) the LMU Section for Neurodiagnostic Applications in Psychiatry (SNAP), focusing on data fusion, disease subtype identification, and clinically scalable algorithms for outcome prediction. The outstanding expertise of this platform will support the DZPG in extracting, understanding, and utilizing predictive information from genetic to psychosocial data layers. Collaborations with other DZPG sites with expertise in computational learning theory and algorithm development could further strengthen the platform’s capacity to model complex psychiatric disorders with ML.

The platform researchers will collaborate with DIFUTURE to build a digital ontology of quantitative psychiatric phenotypes, including clinically relevant measures of disease progression and chronicity in SZ, BPD, and MDD. This phenotypic ontology will lay out a “coordinate system” for the development of a DZPG Model Library that incorporates and disseminates predictive models along the translational chain. Thus, the Library will provide Platform V with well-validated models to facilitate marker-informed clinical trials. Furthermore, predictive signatures hosted in the Library will enable Platforms I and III to explore mechanistic underpinnings and novel modes of action by manipulating key elements of these signatures in appropriate cell and animal model systems and to test them in phase 1 and 2 clinical trials. Finally, adhering fully to open science principles, the Library will enable the DZPG to exchange models with external researchers and thus accelerate the development of reliable precision mental health tools.

The validation opportunities provided by the PriMe Model Library will be supported by our existing longitudinal datasets, which will be made accessible to DZPG-initiated neuromarker research. These datasets will allow to establish the prevalence, relevance, and validity of patient-derived predictive models in large-scale longitudinal cohorts of healthy people (see, e.g., KORA and NAKO studies in the section “Innovative Therapies to Preempt Disease Progression and Chronicity”). Furthermore, our cohorts of patients at different stages of SZ, BPD, and MDD recruited in previous projects (see “Innovative Therapies to Preempt Disease Progression and Chronicity”) or prospectively generated in the DZPG framework (cf. Platform V) will provide researchers with opportunities for external validation and comparative neuroscience across the diagnostic entities covered by the DZPG.

### Platform III: Personalized and Innovative Therapies

We propose to establish a platform in the DZPG for the development of immune- and neuromarker-informed personalized interventions combining existing, repurposed, or novel pharmacological compounds; psychotherapeutic interventions; NTBS; and further treatment modules (e.g., ET, immunomodulatory and neuroprotective therapies). For example, we will use our large-scale and rich databases of exercise interventions in patients with SZ and healthy marathon runners (total N of both >350) and our experience in conducting such trials to (a) enable a better understanding of how exercise induces brain plasticity (reverse translation, Platform I) and (b) facilitate new exercise trials in the DZPG (Platform V). Effective personalization requires transdisciplinary research to (1) better understand multi-level therapeutic mechanisms (Platform I), (2) measure the differential and additive efficacy of existing/repurposed interventions and novel modes of action (Platform I), (3) establish models that inform treatment choices based on the given patient’s estimated response likelihoods and poor outcome risks (Platform II), (4) test novel marker-informed interventions in multi-site clinical trials, and (5) embed this knowledge into routine clinical evidence and care (Platform V). These marker-informed interventions are particularly promising for halting disease progression or even achieving complete recovery in early disease stages.

Psychotherapy plays a major role in primary mental health care and is recommended in current guidelines for SZ, BPD, and MDD. However, any given patient’s access to specific psychotherapeutic methods varies considerably between urban and rural regions. In addition, psychotherapy often needs to be specifically combined with pharmacological interventions to fulfill the different requirements of acute and long-term treatment. As part of Platform III, PriMe researchers aim to individualize psychotherapy based on modules (e.g., trauma-focused, interpersonal, metacognitive, or behavioral activation-based), frequency, duration, and modalities (in person vs. videophone). To this end, predictive studies are needed to identify which patient benefits from which treatment, thus leading to algorithms that optimally sequence and combine psychological and pharmacological interventions.

The infrastructures of the GCBS and the Munich Center for Brain Stimulation will provide a platform for basic research (e.g., tDCS- or rTMS-functional MRI), translational studies (tDCS + cognitive training), IITs (sham-controlled rTMS/tDCS clinical trials), personalized IITs (MRI-guided rTMS/tDCS), and home-treatment applications (tDCS with digital remote monitoring) that uses all available NTBS methods. This long-standing expertise will serve the planned DZPG as a research, intervention, and implementation hub for all aspects of personalized NTBS, i.e., it will (a) develop predictors in proof-of-concept experiments and validate them in RCTs, (b) define and validate cross-disorder paradigms derived from model system findings (e.g., for the treatment of cognitive symptoms), and (c) provide findings from human to the preclinical platforms (reverse translation).

To further exploit the possibilities of physical exercise therapy to improve outcomes and foster recovery across various psychiatric conditions, we will use our large-scale and rich databases of exercise interventions in patients with SZ and healthy marathon runners (total *N* of both >350) and our experience in conducting such trials to (a) enable a better understanding of how exercise induces brain plasticity (reverse translation Platform I) and (b) facilitate new exercise trials in the DZPG (Platform V).

### Platform IV: Ethical, Societal, and Implementation Challenges

Analysis and evaluation of ethics and user involvement in personalized and health technology-based medicine in psychiatry and psychotherapy are core principles of PriMe. Ethically guided personalized profiling based on collating and analyzing a multitude of data from an individual patient implies major ethical challenges regarding health literacy, quality of life, stigmatization, data protection, and allocation of scarce healthcare resources. These challenges arising from the objectives of PriMe touch the ethical principles of autonomy, solidarity, beneficence, non-maleficence, and justice, which govern therapeutic interactions between healthcare professionals and patients and their relatives. The development and implementation of novel health technologies, particularly when these are based on data-driven approaches, require responsible innovation. PriMe will offer its expertise on embedded ethics of medical innovation and health technologies to the DZPG. The integration of ethical principles in the biobehavioral modeling of psychiatric phenotypes will benefit from other DZPG sites that have established track records in neuroethics and philosophy (Figure 5).

Involvement of patients, relatives, and researchers from non-medical disciplines is key for the translation and implementation of research into practice. The PriMe health services research group has pioneering expertise in forming patient advocacy groups. We will offer our expertise in this area to develop standardized procedures that ensure that patients and families are always heard and integrated in the DZPG. A key aspect of such user involvement lies in the development of diagnostic, prognostic, and therapeutic methods that optimally mitigate biases in clinical decision-making. To this end, PriMe will develop standards for implementing personalized profiling tools in in-person and virtual clinical practice. Adaptations of shared decision-making (SDM) for different precision care scenarios will be conceptualized with service users and providers and integrated with other DZPG sites’ expertise in e-Health and telepsychiatry (Figure 5).

### Platform V: Clinical Trials and Evidence-Based Medicine

Another focus of PriMe are IITs, which can be run in our large clinical recruitment network. This approach ensures that the concept of early translation of novel therapies into real-world clinical settings can be readily achieved. Along these lines, PriMe proposes establishing a DZPG Platform for phase II to IV IITs and joining similar efforts for earlier phases of therapeutics development (Figure 5).

Evidence-based medicine and research synthesis are additional strengths of the PriMe consortium, which also hosts the Cochrane Schizophrenia Group. PriMe will produce high-end meta-analyses and support other DZPG partners in this regard. Moreover, it will translate results of meta-analyses into smartphone-based support for SDM, as tested in the ongoing EVENT study. One well-established tool to implement new evidence-based treatments are guidelines. Because of the increasing density of scientific information and unfriendly analogous formats, guidelines are frequently outdated and not sufficiently adopted by clinicians. PriMe researchers are leading or participating in guideline development for SZ, BPD, and MDD and are currently implementing innovative online tools to make these accessible to users, including children and adolescents. PriMe plans to use living guidelines, which continuously integrate newly published studies, to broadly disseminate newest therapeutic evidence to practitioners and patients. In October 2020, SISYPHOS (see the section “Prediction: Translating Understanding Into Precision Medicine Tools”), a collaborative project of PriMe members, received funding to implement such a system for the new German S3 SZ guideline. This project will serve as a blueprint for the development of living German S3 guidelines for other diseases in the DZPG (i.e., interleaved collaboration with platform IV and SDM-guided implementation on Platform V) (Figure 5).

Finally, there is complementarity and collaboration with other German Centers for Health Research. The long-term outcome of mental disorders is determined by risk factors acting throughout the lifespan. Examples of early life risk factors that contribute to mental and somatic disorders in adulthood are infections during pregnancy; birth and delivery complications; vitamin deficiencies; and childhood trauma. Psychiatric and somatic comorbidities emerging during adulthood represent risk factors for chronic disease courses later in life. Both comorbidity clusters are preventable and share common immunological and metabolic mechanisms. PriMe hosts experts on microglia, the resident immune cells of the CNS that play central roles both in neurodevelopmental disorders and neurodegenerative diseases, providing a unique collaborative link with other DZPG centers and between PriMe and CHANCE, the Munich consortium of the DZKJ and the DZNE. Metabolic and inflammatory mechanisms also link PriMe with the health centers for diabetes research (DZD) and heart diseases and CVDs (DZHK).

### Outlook

During the ongoing 6-month Concept Development Phase from September 2021 to February 2022, PriMe will collaborate with all relevant partners to formulate an overarching program and governance structure for the DZPG that involves PriMe and other DZPG sites. To this end, we plan to implement the following steps: Coordinated by the local board of directors, the consortium will establish a PriMe office to support all processes during the Concept Development Phase. A strategic survey of the unmet needs of the main disorders studied in the DZPG will be conducted. In parallel, we will implement a discussion forum for PriMe’s strategic partners, including ECRs and clinician scientists, psychiatrists, psychologists, PCPs, basic researchers, and representatives of patient, caregiver, and advocacy groups. We have held an initial 2-day workshop to agree on the major tasks, milestones, and deliverables for the networking phase and the governance structure and have established a scientific workgroup for each of the four elements of the translational chain (namely understanding and modeling, predictive markers, clinical trials, and implementation). For each of these groups, each DZPG site will delegate a basic scientist, a clinician, an ECR, and a member from a user organization. Each working group has named a spokesperson who is responsible for reporting the progress to the board of directors of the DZPG basis to ensure a continuous build-up of an overarching program resting on the translational chain. Strategic decisions will be achieved with respect to the PriMe site during biweekly meetings of the local board of directors and for the entire DZPG during monthly meetings of the coordinators and co-coordinators at each site. Results from the Concept Development Phase will be presented at the final symposium, and the results of each working group will be published, leading to an overarching program for the DZPG.

## Author Contributions

All authors prepared the manuscript.

## Conflict of Interest

The authors declare that the research was conducted in the absence of any commercial or financial relationships that could be construed as a potential conflict of interest.

## Publisher’s Note

All claims expressed in this article are solely those of the authors and do not necessarily represent those of their affiliated organizations, or those of the publisher, the editors and the reviewers. Any product that may be evaluated in this article, or claim that may be made by its manufacturer, is not guaranteed or endorsed by the publisher.

## Footnotes

1
http://proniapredictors.eu/neurominer/index.html

2
http://proniapredictors.eu/

3
https://zenodo.org/record/4547552#

## References

[1] Kessler RC Angermeyer M Anthony JC Graaf Rd Demyttenaere K Gasquet I Lifetime prevalence and age-of-onset distributions of mental disorders in the World Health Organization’s world mental health survey initiative. *World Psychiatry.* (2007) 6:168–76. 18188442 PMC2174588

[2] Vos T Lim SS Abbafati C Abbas KM Abbasi M Abbasifard M Global burden of 369 diseases and injuries in 204 countries and territories, 1990–2019: a systematic analysis for the Global Burden of Disease Study 2019. *Lancet.* (2020) 396:1204–22. 10.1016/S0140-6736(20)30925-9 33069326 PMC7567026

[3] Runge C Grunze H. Jährliche krankheitskosten bipolarer störungen in Deutschland. *Nervenarzt.* (2004) 75:896–903. 14999464 10.1007/s00115-004-1691-x

[4] Judd LL Akiskal HS Schettler PJ Endicott J Leon AC Solomon DA Psychosocial disability in the course of bipolar I and II disorders: a prospective, comparative, longitudinal study. *Arch Gen Psychiatry.* (2005) 62:1322–30. 10.1001/archpsyc.62.12.1322 16330720

[5] Haefner H an der Heiden W. Course and outcome of schizophrenia. 2nd ed. In: Hirsch SR Weinberger DR editors. *Schizophrenia.* Malden, MA: Blackwell Science (2003). p. 101–41.

[6] Walker ER McGee RE Druss BG. Mortality in mental disorders and global disease burden implications: a systematic review and meta-analysis. *JAMA Psychiatry.* (2015) 72:334–41. 25671328 10.1001/jamapsychiatry.2014.2502 PMC4461039

[7] Lawrence D Hancock KJ Kisely S. The gap in life expectancy from preventable physical illness in psychiatric patients in Western Australia: retrospective analysis of population based registers. *BMJ.* (2013) 346:f2539. 10.1136/bmj.f2539 23694688 PMC3660620

[8] Ringen PA Engh JA Birkenaes AB Dieset I Andreassen OA. Increased mortality in schizophrenia due to cardiovascular disease–a non-systematic review of epidemiology, possible causes, and interventions. *Front Psychiatry.* (2014) 5:137. 10.3389/fpsyt.2014.00137 25309466 PMC4175996

[9] Firth J Siddiqi N Koyanagi A Siskind D Rosenbaum S Galletly C The Lancet Psychiatry Commission: a blueprint for protecting physical health in people with mental illness. *Lancet Psychiatry.* (2019) 6:675–712. 10.1016/S2215-0366(19)30132-4 31324560

[10] Maurus I Hasan A Röh A Takahashi S Rauchmann B Keeser D Neurobiological effects of aerobic exercise, with a focus on patients with schizophrenia. *Eur Arch Psychiatry Clin Neurosci.* (2019) 269:499–515. 10.1007/s00406-019-01025-w 31115660

[11] Falkai P Maurus I Schmitt A Malchow B Schneider-Axmann T Röll L Improvement in daily functioning after aerobic exercise training in schizophrenia is sustained after exercise cessation. *Eur Arch Psychiatry Clin Neurosci.* (2021) 271:1201–3. 10.1007/s00406-021-01282-8 34143287 PMC8429390

[12] Maurus I Hasan A Schmitt A Roeh A Keeser D Malchow B Aerobic endurance training to improve cognition and enhance recovery in schizophrenia: design and methodology of a multicenter randomized controlled trial. *Eur Arch Psychiatry Clin Neurosci.* (2021) 271:315–24. 32748261 10.1007/s00406-020-01175-2 PMC8257533

[13] Leucht S Cipriani A Spineli L Mavridis D Örey D Richter F Comparative efficacy and tolerability of 15 antipsychotic drugs in schizophrenia: a multiple-treatments meta-analysis. *Lancet.* (2013) 382:951–62. 23810019 10.1016/S0140-6736(13)60733-3

[14] Cai N Revez JA Adams MJ Andlauer TFM Breen G Byrne EM Minimal phenotyping yields genome-wide association signals of low specificity for major depression. *Nat Genet.* (2020) 52:437–47. 10.1038/s41588-020-0594-5 32231276 PMC7906795

[15] Klengel T Mehta D Anacker C Rex-Haffner M Pruessner JC Pariante CM Allele-specific FKBP5 DNA demethylation mediates gene-childhood trauma interactions. *Nat Neurosci.* (2013) 16:33–41. 10.1038/nn.3275 23201972 PMC4136922

[16] Halldorsdottir T Piechaczek C Soares de Matos AP Czamara D Pehl V Wagenbuechler P Polygenic risk: predicting depression outcomes in clinical and epidemiological cohorts of youths. *Am J Psychiatry.* (2019) 176:615–25. 10.1176/appi.ajp.2019.18091014 30947532

[17] Dwyer DB Kalman JL Budde M Kambeitz J Ruef A Antonucci LA An investigation of psychosis subgroups with prognostic validation and exploration of genetic underpinnings: the PsyCourse study. *JAMA Psychiatry.* (2020) 77:523–33. 10.1001/jamapsychiatry.2019.4910 32049274 PMC7042925

[18] Prinz M Jung S Priller J. Microglia biology: one century of evolving concepts. *Cell.* (2019) 179:292–311. 10.1016/j.cell.2019.08.053 31585077

[19] Böttcher C Schlickeiser S Sneeboer MAM Kunkel D Knop A Paza E Human microglia regional heterogeneity and phenotypes determined by multiplexed single-cell mass cytometry. *Nat Neurosci.* (2019) 22:78–90. 30559476 10.1038/s41593-018-0290-2

[20] Koutsouleris N Kambeitz-Ilankovic L Ruhrmann S Rosen M Ruef A Dwyer DB Prediction models of functional outcomes for individuals in the clinical high-risk state for psychosis or with recent-onset depression: a multimodal, multisite machine learning analysis. *JAMA Psychiatry.* (2018) 75:1156–72. 10.1001/jamapsychiatry.2018.2165 30267047 PMC6248111

[21] Uher R Tansey KE Rietschel M Heningsberg N Maier W Mors O Common genetic variation and antidepressant efficacy in major depressive disorder: a meta-analysis of three genome-wide pharmacogenetic studies. *Am J Psychiatry.* (2013) 170:207–17. 10.1176/appi.ajp.2012.12020237 23377640 PMC10416089

[22] Bai W Suzuki H Huang J Francis C Wang S Tarroni G A population-based phenome-wide association study of cardiac and aortic structure and function. *Nat Med.* (2020) 26:1654–62. 10.1038/s41591-020-1009-y 32839619 PMC7613250

[23] Stachl C Au Q Schoedel R Gosling SD Harari GM Buschek D Predicting personality from patterns of behavior collected with smartphones. *Proc Natl Acad Sci USA.* (2020) 117:17680–7. 32665436 10.1073/pnas.1920484117 PMC7395458

[24] Riedl V Utz L Castrillón G Grimmer T Rauschecker JP Ploner M Metabolic connectivity mapping reveals effective connectivity in the resting human brain. *Proc Natl Acad Sci USA.* (2016) 113:428–33. 26712010 10.1073/pnas.1513752113 PMC4720331

[25] Avram M Brandl F Cabello J Leucht C Scherr M Mustafa M Reduced striatal dopamine synthesis capacity in patients with schizophrenia during remission of positive symptoms. *Brain.* (2019) 142:1813–26. 10.1093/brain/awz093 31135051

[26] Klaus J Kanton S Kyrousi C Ayo-Martin AC Di Giaimo R Riesenberg S Altered neuronal migratory trajectories in human cerebral organoids derived from individuals with neuronal heterotopia. *Nat Med.* (2019) 25:561–8. 10.1038/s41591-019-0371-0 30858616

[27] Wehr MC Hinrichs W Brzózka MM Unterbarnscheidt T Herholt A Wintgens JP Spironolactone is an antagonist of NRG1-ERBB4 signaling and schizophrenia-relevant endophenotypes in mice. *EMBO Mol Med.* (2017) 9:1448–62. 10.15252/emmm.201707691 28743784 PMC5653977

[28] Hasan A Wobrock T Guse B Langguth B Landgrebe M Eichhammer P Structural brain changes are associated with response of negative symptoms to prefrontal repetitive transcranial magnetic stimulation in patients with schizophrenia. *Mol Psychiatry.* (2017) 22:857–64. 10.1038/mp.2016.161 27725655

[29] Maurus I Hasan A Schmitt A Roeh A Keeser D Malchow B Correction to: aerobic endurance training to improve cognition and enhance recovery in schizophrenia: design and methodology of a multicenter randomized controlled trial. *Eur Arch Psychiatry Clin Neurosci.* (2021) 271:1405–6. 10.1007/s00406-021-01289-1 34226950 PMC8429360

[30] Böge K Karnouk C Hahn E Schneider F Habel U Banaschewski T Mental health in refugees and asylum seekers (MEHIRA): study design and methodology of a prospective multicentre randomized controlled trail investigating the effects of a stepped and collaborative care model. *Eur Arch Psychiatry Clin Neurosci.* (2020) 270:95–106. 10.1007/s00406-019-00991-5 30796528

[31] Huhn M Nikolakopoulou A Schneider-Thoma J Krause M Samara M Peter N Comparative efficacy and tolerability of 32 oral antipsychotics for the acute treatment of adults with multi-episode schizophrenia: a systematic review and network meta-analysis. *Lancet.* (2019) 394:939–51. 10.1016/s0140-6736(19)31135-3 31303314 PMC6891890

[32] Brouwer RM Klein M Grasby KL Schnack HG Jahanshad N Teeuw J Dynamics of brain structure and its genetic architecture over the lifespan. *bioRxiv* [Preprint]. (2020). 10.1101/2020.04.24.031138

[33] Takahashi S Keeser D Rauchmann B-S Schneider-Axmann T Keller-Varady K Maurus I Effect of aerobic exercise combined with cognitive remediation on cortical thickness and prediction of social adaptation in patients with schizophrenia. *Schizophr Res.* (2020) 216:397–407. 10.1016/j.schres.2019.11.004 31806522

[34] Takahashi S Keeser D Rauchmann B-S Schneider-Axmann T Keller-Varady K Maurus I Effect of aerobic exercise on cortical thickness in patients with schizophrenia–a dataset. *Data Brief.* (2020) 30:105517. 10.1016/j.dib.2020.105517 32395575 PMC7210414

[35] Fiske A Henningsen P Buyx A. Your robot therapist will see you now: ethical implications of embodied artificial intelligence in psychiatry, psychology, and psychotherapy. *J Med Internet Res.* (2019) 21:5. 10.2196/13216 31094356 PMC6532335

[36] Collins FS Rossant J Wurst W. A mouse for all reasons. *Cell.* (2007) 128:9–13. 10.1016/j.cell.2006.12.018 17218247

[37] Linseisen J Peters A Pischon T Willich S Keil T Boeing H The German National Cohort: aims, study design and organization. *Eur J Epidemiol.* (2014) 29:371–82. 10.1007/s10654-014-9890-7 24840228 PMC4050302

[38] Heinrich J Brüske I Cramer C Hoffmann U Schnappinger M Schaaf B GINIplus and LISAplus–design and selected results of two German birth cohorts about natural course of atopic diseases and their determinants. *Allergol Select.* (2017) 1:85–95. 10.5414/ALX01455E 30402607 PMC6040001

[39] Schuster F Holzhüter F Heres S Hamann J. Caregiver involvement in psychiatric inpatient treatment–a representative survey among triads of patients, caregivers and hospital psychiatrists. *Epidemiol Psychiatr Sci.* (2020) 29:129. 10.1017/S2045796020000426 32438939 PMC7264704

[40] Hamann J Heres S Seemann U Beitinger R Spill B Kissling W. Effects of an integrated care program for outpatients with affective or psychotic disorders. *Psychiatry Res.* (2014) 217:15–9. 10.1016/j.psychres.2014.02.005 24656902

[41] Riedl L Blank D Kohl M Lang A Kehl V Brieger P Return-to-work-experts for inpatient treatment of patients with mental illnesses– a proof-of-concept-study (RETURN): the study protocol. *BMC Psychiatry.* (2020) 20:177. 10.1186/s12888-020-02504-4 32306925 PMC7168961

[42] Hasan A Roeh A Leucht S Langguth B Hansbauer M Oviedo-Salcedo T Add-on spironolactone as antagonist of the NRG1-ERBB4 signaling pathway for the treatment of schizophrenia: study design and methodology of a multicenter randomized, placebo-controlled trial. *Contemp Clin Trials Commun.* (2020) 17:100537. 10.1016/j.conctc.2020.100537 32072071 PMC7013159

[43] Koutsouleris N Wobrock T Guse B Langguth B Landgrebe M Eichhammer P Predicting response to repetitive transcranial magnetic stimulation in patients with schizophrenia using structural magnetic resonance imaging: a multisite machine learning analysis. *Schizophr Bull.* (2018) 44:1021–34. 10.1093/schbul/sbx114 28981875 PMC6101524

[44] Zannas AS Jia M Hafner K Baumert J Wiechmann T Pape JC Epigenetic upregulation of FKBP5 by aging and stress contributes to NF-κB-driven inflammation and cardiovascular risk. *Proc Natl Acad Sci USA.* (2019) 116:11370–9. 10.1073/pnas.1816847116 31113877 PMC6561294

[45] Zannas AS Wiechmann T Gassen NC Binder EB. Gene-stress-epigenetic regulation of FKBP5: clinical and translational implications. *Neuropsychopharmacology.* (2016) 41:261–74. 10.1038/npp.2015.235 26250598 PMC4677131

